# Renal and survival benefits of seventeen prescribed Chinese herbal medicines against oxidative-inflammatory stress in systemic lupus erythematosus patients with chronic kidney disease: a real-world longitudinal study

**DOI:** 10.3389/fphar.2023.1309582

**Published:** 2024-01-03

**Authors:** Hsiao-Tien Chen, Chien-Hsueh Tung, Ben-Hui Yu, Ching-Mao Chang, Yi-Chun Chen

**Affiliations:** ^1^ Department of Chinese Medicine, Chi Mei Medical Center, Tainan City, Taiwan; ^2^ Division of Allergy, Immunology and Rheumatology, Department of Internal Medicine, Dalin Tzu Chi Hospital, Buddhist Tzu Chi Medical Foundation, Chiayi, Taiwan; ^3^ School of Medicine, Tzu Chi University, Hualien, Taiwan; ^4^ Department of Radiation Oncology, Dalin Tzu Chi Hospital, Buddhist Tzu Chi Medical Foundation, Chiayi, Taiwan; ^5^ Center for Traditional Medicine, Taipei Veterans General Hospital, Taipei, Taiwan; ^6^ Institute of Traditional Medicine, National Yang Ming Chiao Tung University, Taipei, Taiwan; ^7^ School of Medicine, College of Medicine, National Yang Ming Chiao Tung University, Taipei, Taiwan; ^8^ Division of Nephrology, Department of Internal Medicine, Dalin Tzu Chi Hospital, Buddhist Tzu Chi Medical Foundation, Chiayi, Taiwan

**Keywords:** renoprotective Chinese herbal medicines, systemic lupus erythematosus, lupus nephritis, CKD, oxidative-inflammatory stress, ESRD, all-cause mortality, network analysis

## Abstract

**Background:** Systemic lupus erythematosus (SLE) significantly links to LN, a type of CKD with high mortality despite modern Western treatments. About 70% of SLE patients develop LN, and 30% advance to end-stage renal disease (ESRD). Concerns about glucocorticoid side effects and LN worsening due to oxidative stress prompt alternative treatment searches. In Taiwan, over 85% of SLE patients opt for complementary methods, especially Chinese herbal medicine (CHM). We pinpointed seventeen CHMs for SLE (PRCHMSLE) with antioxidative and anti-inflammatory properties from national health insurance data (2000–2017). Our primary aim was to assess their impact on renal and survival outcomes in SLE patients progressing to CKD (SLE-CKD), with a secondary focus on the risks of hospitalization and hyperkalemia.

**Methods:** We established a propensity-matched cohort of 1,188 patients with SLE-CKD, comprising 594 PRCHMSLE users and 594 nonusers. We employed Cox proportional hazards models and restricted mean survival time (RMST) analyses to assess the renal and survival outcomes of PRCHMSLE users. Moreover, we performed pooling and network analyses, specifically focusing on the renal effects linked to PRCHMSLE.

**Results:** PRCHMSLE use was associated with decreased adjusted hazard ratios for ESRD (0.45; 95% confidence interval, 0.25–0.79, *p* = 0.006), all-cause mortality (0.56; 0.43–0.75, *p* < 0.0001), non-cardiovascular mortality (0.56; 0.42–0.75, *p* < 0.0001), and hospitalization (0.72; 0.52–0.96, *p* = 0.009). Hyperkalemia risk did not increase. Significant differences in RMST were observed: 0.57 years (95% confidence interval, 0.19–0.95, *p* = 0.004) for ESRD, 1.22 years (0.63–1.82, *p* < 0.0001) for all-cause mortality, and 1.21 years (0.62–1.80, *p* < 0.0001) for non-cardiovascular mortality, favoring PRCHMSLE use. Notably renoprotective PRCHMSLE included Gan-Lu-Ying, *Anemarrhena asphodeloides* Bunge [Asparagaceae; Rhizoma Anemarrhenae] (Zhi-Mu), *Rehmannia glutinosa* (Gaertn.) DC. [Orobanchaceae; Radix Rehmanniae] (Sheng-Di-Huang), Jia-Wei-Xiao-Yao-San, and *Paeonia suffruticosa* Andr. [Paeoniaceae; Cortex Moutan] (Mu-Dan-Pi). Network analysis highlighted primary treatment strategies with central components like Liu-Wei-Di-Huang-Wan, *Paeonia suffruticosa* Andr. [Paeoniaceae; Cortex Moutan] (Mu-Dan-Pi), *Anemarrhena asphodeloides* Bunge [Asparagaceae; Rhizoma Anemarrhenae] (Zhi-Mu), *Rehmannia glutinosa* (Gaertn.) DC. [Orobanchaceae; Radix Rehmanniae] (Sheng-Di-Huang), and Zhi-Bai-Di-Huang-Wan.

**Conclusion:** This work underscores the pronounced renal and survival benefits associated with the seventeen PRCHMSLE in the treatment of SLE-CKD, concurrently mitigating the risks of hospitalization and hyperkalemia. This highlights their potential as alternative treatment options for individuals with this condition.

## 1 Introduction

Systemic lupus erythematosus (SLE) is an autoimmune disease characterized by multiorgan inflammation and is a significant cause of lupus nephritis (LN) (Davidson, 2016). An alarming 70% of individuals with SLE suffer from lupus nephritis, and up to 30% of lupus patients progress to ESRD, making it the most severe and frequent manifestation of the disease (Maroz and Segal, 2013). This impact is characterized by the glomerular deposition of immune complexes, which subsequently triggers an inflammatory response (Davidson, 2016). Despite advancements in immunosuppressive therapies, there has not been a significant decline in the progression to ESRD or in mortality rates in recent decades. The prognosis for these patients is grim; those who progress to ESRD have a staggering 26-fold increased risk of mortality (Yap et al., 2012).

Oxidative stress and inflammation are critical factors in the pathogenesis of SLE, notably aggravating LN by initiating immune complex deposition in the glomeruli, promoting inflammatory cell recruitment, and causing progressive fibrosis (Bona et al., 2020; Justiz Vaillant et al., 2023). Glucocorticoids inhibit many of the initial events in the inflammatory response, significantly improving the prognosis for those with LN (Coutinho and Chapman, 2011). However, both short-term high doses and cumulative exposure to glucocorticoids can lead to undesirable side effects, including cardiovascular events, peptic ulcers, sleep disorders, weight gain, and osteoporosis (Mejia-Vilet and Ayoub, 2021). As a result, a recent cross-sectional study revealed that over 85% of Taiwanese patients with SLE regularly employ complementary therapies (Lu et al., 2022).

Chinese herbal medicine (CHM) is a prominent complementary medicinal system worldwide, with its clinical practices dating back thousands of years (Liu et al., 2022). Previous research has shown reduced risks of ESRD and overall mortality in patients with advanced chronic kidney disease (CKD) (Chen et al., 2022) and advanced diabetic kidney disease (Guo et al., 2021). There are also reports of decreased risks of overall mortality (Ma et al., 2016), LN (Chang et al., 2017), and cardiovascular disease (Yu and Hsieh, 2021) in SLE patients using CHM. Evidence suggests that seventeen potentially renoprotective CHMs for SLE (PRCHMSLE), which counteract oxidative-inflammatory stress collectively, are acknowledged as complementary treatment options for SLE (Huang et al., 2016; Jiang et al., 2020; Wang et al., 2021). These 17 PRCHMSLE are *Rehmannia glutinosa* (Gaertn.) DC. [Orobanchaceae; Radix Rehmanniae] (Sheng-Di-Huang), *Anemarrhena asphodeloides* Bunge [Asparagaceae; Rhizoma Anemarrhenae (Zhi-Mu), *Paeonia lactiflora* Pall. [Paeoniaceae; Radix Paeoniae Alba] (Bai Shao), *Paeonia suffruticosa* Andr. [Paeoniaceae; Cortex Moutan (Mu-Dan-Pi), *Salvia miltiorrhiza* Bge. [Lamiaceae; Radix Salviae Miltiorrhizae (Dan-Shan), *Paeonia lactiflora* Pall. var. rubra [Paeoniaceae; Radix Paeoniae Rubra] (Chi-Shao), *Lithospermum erythrorhizon* Siebold & Zucc. [Boraginaceae; Radix Lithospermi] (Zi-Cao), *Artemisia annua* L. [Asteraceae; Herba Artemisiae Annuae (Qing-Hao), *Hedyotis diffusa* Willd. [Rubiaceae; Herba Hedyotis] (Bai-Hua-She-She-Cao), *Scutellaria barbata* D. Don [Lamiaceae; Herba Scutellariae Barbatae (Ban-Zhi-Lian), Zhi-Bai-Di-Huang-Wan, Liu-Wei-Di-Huang-Wan, Gan-Lu-Ying, Qin-Jiao-Bie-Jia-Tang, Jia-Wei-Xiao-Yao-San, Yin-Qiao-San, and Gui-Zhi-Shao-Yao-ZhiMu-Tang (Supplementary Table S1). However, there exists a gap in large-scale evidence assessing the effects of these 17 PRCHMSLE on ESRD and mortality outcomes, as well as their pooling effect and a network core pattern analysis on ESRD outcomes in SLE patients progressing to CKD. Given that Taiwan’s National Health Insurance (NHI) covers prescribed CHMs that are free from aristolochic acid (Chen et al., 2022), we utilized NHI longitudinal claims data from 2000 to 2017 to bridge this knowledge gap.

## 2 Materials and methods

### 2.1 Study design and data source

The retrospective cohort study examined 2 million de-identified claims from Taiwan’s 2005 Longitudinal Generation Tracking Database (LGTD 2005) spanning the years 2000–2017. Given the nature of the data, informed consent was not required, and the study received an exemption from a full review by the Institutional Review Board of the Dalin Tzu Chi Hospital (B10804001). The LGTD2005 was randomly selected from the entirety of beneficiaries under Taiwan’s NHI program. Further details about Taiwan’s LGTD2005 and the NHI program are discussed in our previous research (Chen et al., 2015a; Chen et al., 2022; Chen et al., 2023). The LGTD2005 employs the ICD-9-CM and, starting in 2016, the ICD-10-CM diagnostic codes for disease identification. While it provides extensive data on medication and herbal treatments, it does not include information on laboratory results or lifestyle factors.

### 2.2 Study population (SLE patients progressing to CKD [patients with SLE-CKD])

**FIGURE 1:**
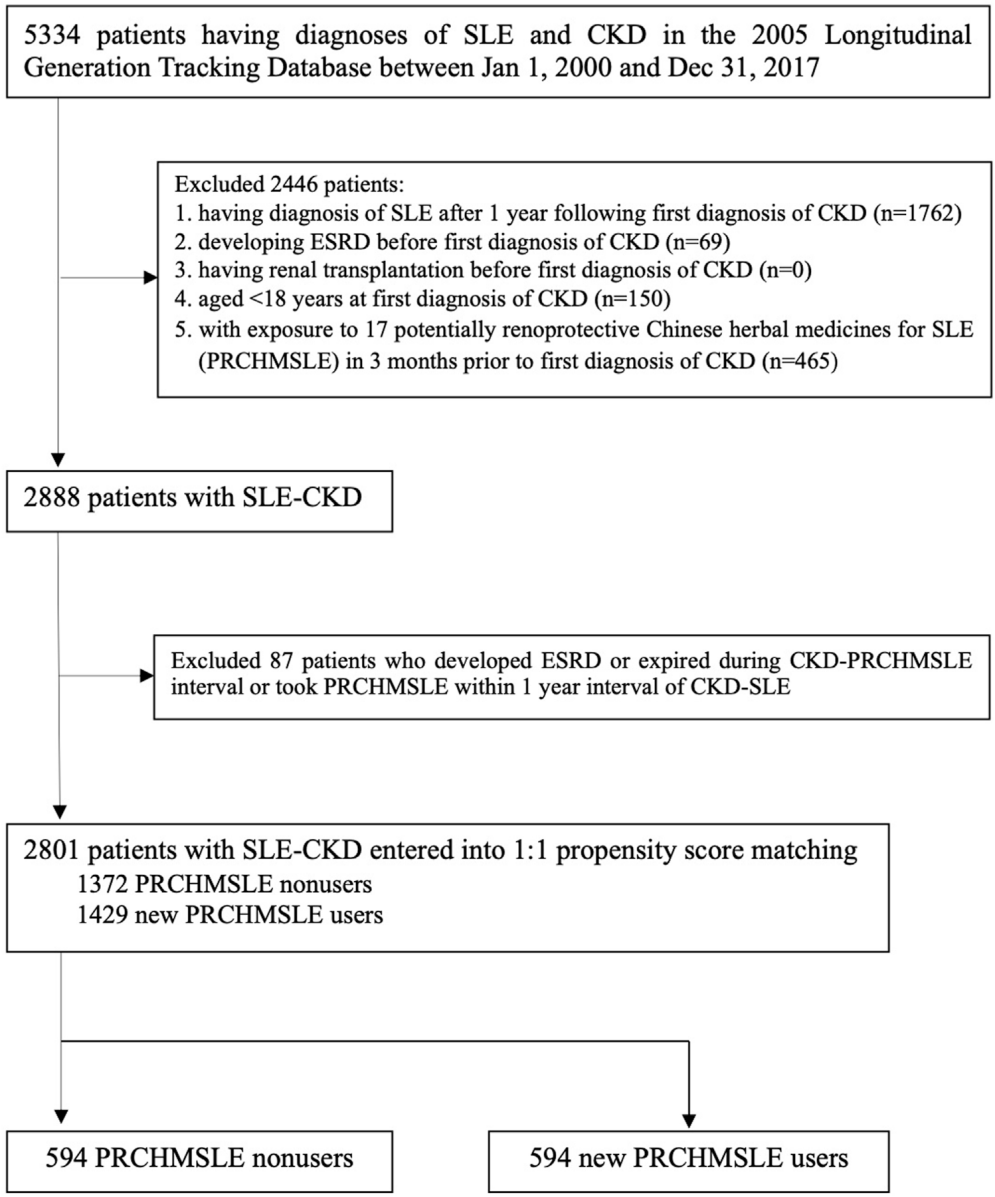
Flowchart for selecting *systemic lupus erythematosus* (SLE) patients progressing to chronic kidney disease (CKD).

The study cohort comprised 5,334 patients diagnosed with both SLE and CKD from 1 January 2000, to 31 December 2017 (Figure 1). We excluded patients under 18 years at the time of their initial CKD diagnosis, patients diagnosed with SLE (based on ICD 9/10-CM codes) more than a year after their first CKD diagnosis, patients who developed ESRD or underwent renal transplantation before their initial CKD diagnosis, and patients who were exposed to the 17 PRCHMSLE within 3 months prior to their first CKD diagnosis. After these exclusions, 2888 SLE patients progressing to CKD (SLE-CKD) were enrolled from 2000 to 2017.

### 2.3 Exposure to seventeen potentially renoprotective CHMs for SLE (PRCHMSLE)

SLE patients who received at least one of any 17 PRCHMSLE (Supplementary Table S1) after CKD diagnosis during the study period were categorized as PRCHMSLE users, aligning with the user definition employed in previous studies (Chang et al., 2017; Chen et al., 2022). The remaining SLE patients who never used any 17 PRCHMSLE after CKD diagnosis during the study period were defined as nonusers. Considering the exposure to the 17 PRCHMSLE post the first CKD diagnosis, and excluding patients who developed ESRD, those who passed away during the CKD-PRCHMSLE interval, and those who began using PRCHMSLE within a year of the CKD-SLE diagnosis, we identified 2,801 patients with SLE-CKD from 2000 to 2017. Of this cohort, 1,429 (51%) were new PRCHMSLE users, and 1,372 (49%) were nonusers.

### 2.4 Covariates

We assessed several variables, including age, sex, Charlson comorbidity index (an indicator of overall disease burden), and frequency of medical visits (to address potential detection bias). Baseline comorbidities considered were diabetes (defined by ICD-9/10- CM codes or the use of antihyperglycemic drugs), hypertension (defined by ICD-9/10-CM codes or antihypertensives), hyperlipidemia (defined by ICD-9/10-CM codes or antilipidemic drugs) (Chen et al., 2022), rheumatoid arthritis, Sjögren’s syndrome, and Raynaud’s disease (all defined by ICD-9/10-CM codes) (Chang et al., 2017). Additionally, we evaluated the use of three potentially confounding medications—non-steroidal anti-inflammatory drugs, steroids (including prednisolone and methylprednisolone), and other medications apart from steroids (such as cyclophosphamide, azathioprine, hydroxychloroquine, and mycophenolate mofetil) (Chang et al., 2017)—within the year prior to the index date.

### 2.5 Propensity score matching

Propensity score method was employed to mitigate confounding related to the indication of PRCHMSLE utilization. The propensity score, indicating the likelihood of using PRCHMSLE, was calculated using the logistic regression that was built on all covariates (age per year, sex, comorbidities, Charlson comorbidity index, number of medical visits, and confounding drugs) to adjust for the baseline differences between PRCHMSLE users and nonusers. Propensity score matching was performed using the nearest-neighbor approach without replacement and employed a caliper value of 0.0001 to ensure precision throughout the matching process (Chen et al., 2022). For each PRCHMSLE user, a propensity score-matched nonuser was chosen. To mitigate immortal bias (Chen et al., 2022; Chen et al., 2023), it was verified that each matched nonuser was alive when PRCHMSLE usage began. The index date for PRCHMSLE users was marked as the exact day when PRCHMSLE therapy was initiated, confirming that PRCHMSLE users had lived from the start of CKD up to this index date. For nonusers, the index date was aligned to coincide with the exact day of PRCHMSLE initiation by the user.

### 2.6 Study outcomes and follow-up

The primary outcomes of this study were ESRD, all-cause mortality, cardiovascular mortality, and non-cardiovascular mortality. Participants were tracked from their index date until the event of ESRD or other censoring occurrences, such as death or the study’s conclusion on 31 December 2017, whichever was earlier. ESRD was identified based on the acquisition of a catastrophic illness certificate for long-term dialysis (Chen et al., 2015a; Chen et al., 2022; Chen et al., 2023). Mortality was ascertained by a patient’s withdrawal from the NHI program (Chen et al., 2015a; Chen et al., 2022; Chen et al., 2023). In the mortality outcome analysis, patients were consistently observed up to their time of death. Cardiovascular mortality included deaths attributed to primary diagnoses such as coronary heart disease, stroke, peripheral vascular disease, and heart failure, as classified by ICD 9/10-CM codes (Yu et al., 2022). If ESRD events occurred earlier during the follow-up, they were not treated as censoring points (Huang et al., 2022). The secondary outcomes investigated in this study were concentrated on assessing the risks of hospitalization and hyperkalemia. Hospitalization rates were considered as a proxy for glucocorticoid side effects (Peterson et al., 2021), while the presence of both SLE (Lee et al., 1988) and CKD (Chen et al., 2022) heightened the risk of hyperkalemia. Incidents of hospitalization and hyperkalemia were monitored throughout the study period, with the latter being identified through the presence of ICD-9/10-CM codes for hyperkalemia or the utilization of potassium-lowering agents (Chen et al., 2022).

### 2.7 Pooling effects of 17 PRCHMSLE

We evaluated the pooling effects of the 17 PRCHMSLE on ESRD occurrence and identified the top five agents demonstrating the most robust renoprotective effects.

### 2.8 Network analysis

To identify the primary patterns of CHM use in treating SLE, we utilized the open-source tool NodeXL (http://nodexl.codeplex.com/), as detailed in our previous research (Chang et al., 2015). Every selected CHM combination was incorporated into this network analysis. Within the network diagram, relationships between a CHM and its associated prescription were illustrated using lines of varying thickness, from 1 to 5, with thicker lines representing more common prescription patterns. This method effectively emphasized the dominant trends in the prescription of these renoprotective CHMs throughout our study.

### 2.9 Statistical analyses

Disparities in baseline characteristics between PRCHMSLE users and nonusers were assessed using the standardized mean difference approach. A value of <0.1 signified minimal distinction between the two groups after propensity score matching (Chen et al., 2022). Death prior to ESRD occurrence was considered a competing risk event (Chen et al., 2015a). For the cumulative incidence of ESRD, the calculation and comparison in data with competing risk were conducted using modified Kaplan-Meier and Gray’s methods (Chen et al., 2015a). In analyzing all-cause mortality, the Kaplan-Meier method was employed. We assessed differences in the complete time-to-event distributions between the study groups by utilizing a modified log-rank test for ESRD and a log-rank test for all-cause mortality.

We computed the incidence rates per 1,000 person-years for the study outcomes in both groups. The assumption of proportional hazards was confirmed using a log (-log (survival)) plot against the log of survival time, which demonstrated no violations. The primary and secondary study outcomes were assessed using the Cox proportional hazard model. By comparing PRCHMSLE users with nonusers, we estimated adjusted hazard ratios (aHRs) with their associated 95% confidence intervals (CIs). These estimates considered all covariates mentioned in Table 1 and considered competing mortality when evaluating the risk of ESRD (Chen et al., 2015a).

**TABLE 1:** Baseline characteristics of study cohorts by use of 17 potentially renoprotective Chinese herbal medicines for SLE (PRCHMSLE) in patients with SLE-CKD.

Variable	Propensity-matched patients with SLE-CKD		
	Users (n = 594)	Nonusers (n = 594)	SMD
Sex, *n* (%)			0.018
Men	102 (17.2)	106 (17.9)	
Women	492 (82.8)	488 (82.2)	
Age (year), *n* (%)			0.021
≤35	155 (26.1)	154 (25.9)	
35–50	176 (29.6)	177 (29.8)	
50–65	154 (25.9)	151 (25.4)	
>65	109 (18.4)	112 (18.9)	
Mean (SD)	48.3 (16.9)	48.7 (17.7)	
Comorbidities, *n* (%)			
Diabetes	67 (11.3)	61 (10.3)	0.033
Hypertension	295 (49.7)	288 (48.5)	0.024
Hyperlipidemia	93 (15.7)	86 (14.5)	0.033
Rheumatoid arthritis	26 (4.4)	21 (3.5)	0.043
*Sjögren’s* syndrome	19 (3.2)	25 (4.2)	0.053
Raynaud’s disease	6 (1.0)	5 (0.8)	0.018
Charlson comorbidity index, *n* (%)			0.017
≤1	398 (67.0))	405 (68.1)	
1–2	110 (18.5)	108 (18.2)	
2–4	58 (9.8)	54 (9.1)	
≥4	28 (4.7)	27 (4.6)	
Mean (±SD)	1.23 (1.2)	1.21 (1.2)	
No. of medical visits, *n* (%)			0.115
≤12	190 (32.0)	201 (33.8)	
12–24	181 (30.5)	185 (31.2)	
>24	223 (37.5)	208 (35.0)	
Mean (SD)	23.3 (18.6)	21.3 (16.6)	
Confounding drugs, *n* (%)			
NSAID	484 (81.5)	481 (81.0)	0.013
Steroids^†^	43 (7.2)	39 (6.6)	0.026
Drugs other than steroids^††^	15 (2.5)	13 (2.2)	0.022

Abbreviations: SLE, systemic lupus erythematosus; CKD, chronic kidney disease; SD, standard deviation; NSAID, non-steroidal anti-inflammatory drug; SMD, standardized mean difference.

^†^ Include prednisolone and methylprednisolone.

^††^Include cyclophosphamide, azathioprine, hydroxycholorquine, and mycopenolate mofetil.

Complementary restricted mean survival time (RMST) analysis was utilized specifically to evaluate the primary study outcomes. RMST, defining the area beneath the survival curve up to a given (restricted) time, was employed as an alternative to the conventional Cox analysis (Perego et al., 2020). The difference in RMST quantified the delay in achieving a specific outcome during a given interval and depicted the disparity between the areas under the survival curves for the intervention and control groups. Hence, the RMST difference was instrumental in assessing the clinical significance of an advantage, separate from the relative treatment effects highlighted by HRs. We gauged the RMST differences by contrasting the areas under the survival curves between PRCHMSLE users and nonusers. A positive RMST difference was in favor of PRCHMSLE treatment, indicating an average delay in achieving the study outcomes among the two groups. Additionally, we reviewed the 15-year RMST difference, along with its associated 95% CI, for the study outcomes between the two groups. This juxtaposition enriched the insights beyond the aHR evaluations and facilitated a holistic comprehension of the results.

We also employed Poisson regression to estimate the adjusted incident rate ratio of hyperkalemia associated with PRCHMSLE use. Recurrent episodes of hyperkalemia were classified as a single prolonged event when they transpired within 28 days of each other, whereas episodes occurring at least 28 days apart were treated as distinct events (Chen et al., 2022).

Statistical analyses were conducted using SAS software (version 9.4; SAS Institute, Inc., Cary, N.C., USA). A statistical significance was determined if the 95% CI for aHRs did not encompass 1, or when the 95% CI for the difference in RMST did not contain 0. A two-tailed *p*-value less than 0.05 was considered statistically significant.

### 2.10 Sensitivity analyses

To enhance the reliability of our primary findings, we carried out three sensitivity analyses. First, the PRCHMSLE-usage group was redefined based on cumulative usage exceeding 30 and 60 days. Second, the risk assessment for study outcomes was reassessed by excluding CKD patients who either passed away or advanced to ESRD within 30, 60, or 90 days post the index date. Lastly, subgroup analyses were conducted considering baseline characteristics.

## 3 Results

### 3.1 Baseline characteristics

After propensity score matching, a balanced distribution of all baseline characteristics was observed between 594 PRCHMSLE users and 594 nonusers in the SLE-CKD patient group (Table 1; Supplementary Figure S1). This resulted in a discernible differentiation between the matched cohorts, evidenced by a c-index of 0.65 and a Hosmer–Lemeshow test *p*-value of 0.39, suggesting a satisfactory model fit.

### 3.2 Association between 17 PRCHMSLE and the primary study outcomes

**FIGURE 2:**
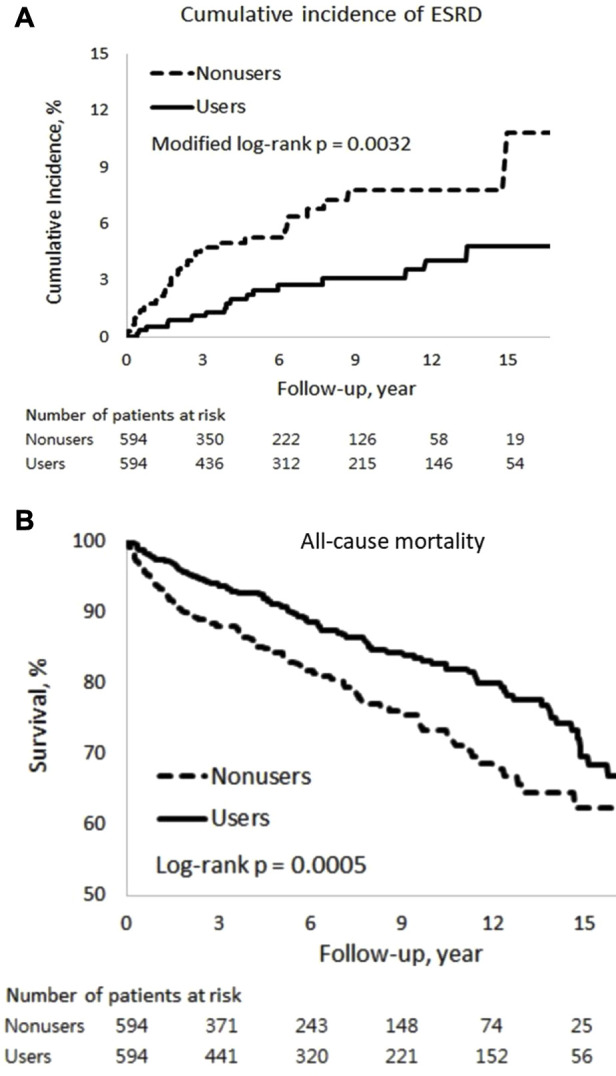
Cumulative incidences of **(A)** end-stage renal disease (ESRD) and **(B)** all-cause mortality among PRCHMSLE users and nonusers.

Among patients with SLE-CKD, 50 individuals (4.2%) progressed to ESRD, while 207 (17.4%) experienced all-cause deaths, with 5 attributed to cardiovascular mortality and 202 to non-cardiovascular mortality (Table 2). The 15-year cumulative incidences of ESRD (*p* = 0.0032) in the presence of competing mortality and all-cause mortality (*p* = 0.0005) were markedly lower for PRCHMSLE users compared to nonusers (Figure 2). After adjusting for all covariates, PRCHMSLE use in patients with SLE-CKD was significantly correlated with decreased risks of ESRD (aHR: 0.45; 95% CI: 0.25–0.79, *p* = 0.006), all-cause mortality (aHR: 0.56; 95% CI: 0.43–0.75, *p* < 0.0001), cardiovascular mortality (aHR: 0.69; 95% CI: 0.11–4.27, *p* = 0.69), and non-cardiovascular mortality (aHR: 0.56; 95% CI: 0.42–0.75, *p* < 0.0001). Over a 15-year span, compared to nonuse, PRCHMSLE use was linked to a delay of 0.57 (95% CI: 0.19–0.95, *p* = 0.004), 1.22 years (95% CI: 0.63–1.82, *p* < 0.0001), 0.02 years (95% CI: 0.10–0.14, *p* = 0.74), and 1.21 years (95% CI: 0.62–1.80, *p* < 0.0001) in the onset of ESRD, all-cause mortality, cardiovascular mortality, and non-cardiovascular mortality, respectively. Considering a type I error α of 0.05, an event rate of 0.034 per year for the nonuser group, a median follow-up of 5.4 years, a censoring rate of 0.96, and a user-to-nonuser ratio of 1:1, we would need 490 participants in both user and non-user groups to achieve a power (1-β) of 0.9, sufficient to detect a 50% shift in the hazard ratio. Given our sample of 594 individuals in each category and an aHR of 0.45, our study displays a test power exceeding 0.9.

**TABLE 2:** 15-year-restricted mean survival time (RMST) and Cox proportional hazards model analyses on study outcomes among propensity score–matched PRCHMSLE users and PRCHMSLE nonusers.

Outcomes	Events (%)		Estimated event rate (events/1,000 person-years)		RMST (95% CI), tau = 15 years					
	Users	Nonusers	Users	Nonusers	Users	Nonusers	Difference, year (95% CI)	*p-value*	Estimated aHR (95% CI)	*p-value*
ESRD	17 (2.9)	33 (5.6)	3.9	10.6	14.57 (14.39, 14.78)	13.99 (13.69, 14.34)	0.57 (0.19, 0.95)	0.004	0.45 (0.25, 0.79)	0.006
All-cause mortality	92 (15.5)	115 (19.4)	20.7	34.1	12.99 (12.65, 13.38)	11.8 (11.37, 12.31)	1.22 (0.63, 1.82)	<0.0001	0.56 (0.43, 0.75)	<0.0001
Cardiovascular mortality	2 (0.34)	3 (0.51)	0.45	0.89	14.9 (14.8, 15.1)	14.9 (14.8, 15.0)	0.02 (−0.10, 0.14)	0.74	0.69 (0.11, 4.27)	0.69
Non-cardiovascular mortality	90 (15.2)	112 (18.9)	20.3	33.2	13.0 (12.6, 13.4)	11.9 (11.4, 12.4)	1.21 (0.62, 1.80)	<0.0001	0.56 (0.42, 0.75)	<0.0001

Abbreviations: PRCHMSLE, potentially renoprotective Chinese herbal medicines for systemic lupus erythematosus; ESRD, end-stage renal disease; aHR, adjusted hazard ratio; CI, confidence interval.

Adjusted for all covariates (age per year, sex, comorbidities, number of medical visits, Charlson comorbidity index, confounding drugs), and competing risk for ESRD.

### 3.3 Association between 17 PRCHMSLE and the secondary study outcomes

PRCHMSLE use was associated with lower risks of hospitalization (aHR: 0.72; 95% CI: 0.56–1.92, *p* = 0.009) (Supplementary Table S5). Moreover, PRCHMSLE use was not associated with an elevated risk of hyperkalemia (adjusted incidence rate ratio: 0.74; 95% CI: 0.49–1.10, *p* = 0.13) (Supplementary Table S6).

### 3.4 Top five renoprotective effects among the 17 PRCHMSLE

**FIGURE 3:**
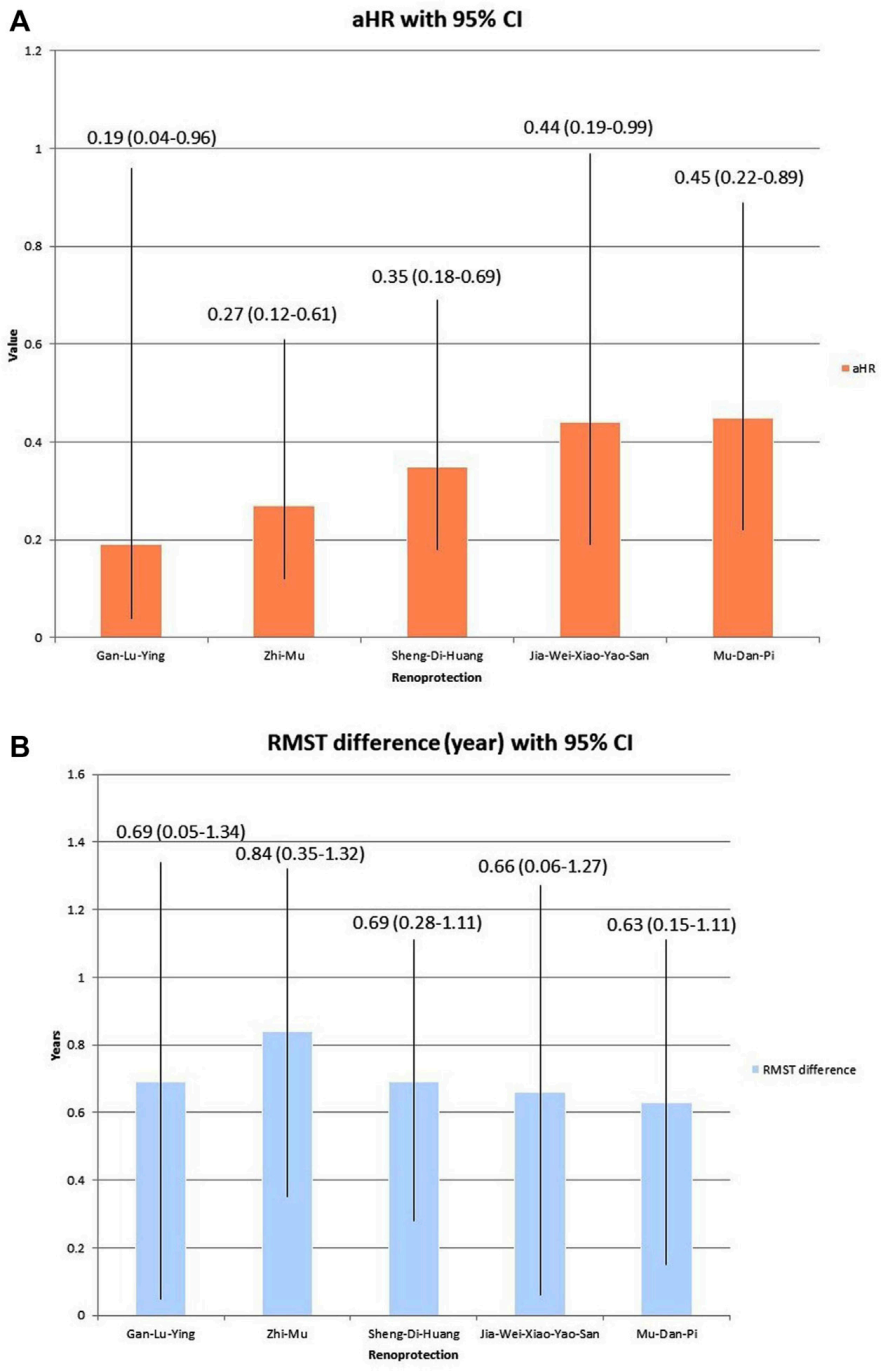
Top five renoprotection in the pooling effect of 17 potentially renoprotective Chinese herbal medicines for SLE (PRCHMSLE), analyzed through **(A)** adjusted hazard ratios (aHRs) of end-stage renal disease and **(B)** restricted mean survival time (RMST) differences in end-stage renal disease. Abbreviation: CI, confidence interval.

Utilizing both the Cox model (Figure 3A) and RMST (Figure 3B) analyses, the top five PRCHMSLE formulas that exhibited the most pronounced renoprotective effects through pooling were Gan-Lu-Ying, *Anemarrhena asphodeloides* Bunge [Asparagaceae; Rhizoma Anemarrhenae] (Zhi-Mu), *Rehmannia glutinosa* (Gaertn.) DC. [Orobanchaceae; Radix Rehmanniae] (Sheng-Di-Huang), Jia-Wei-Xiao-Yao-San, and *Paeonia suffruticosa* Andr. [Paeoniaceae; Cortex Moutan] (Mu-Dan-Pi).

### 3.5 Network analysis

**FIGURE 4:**
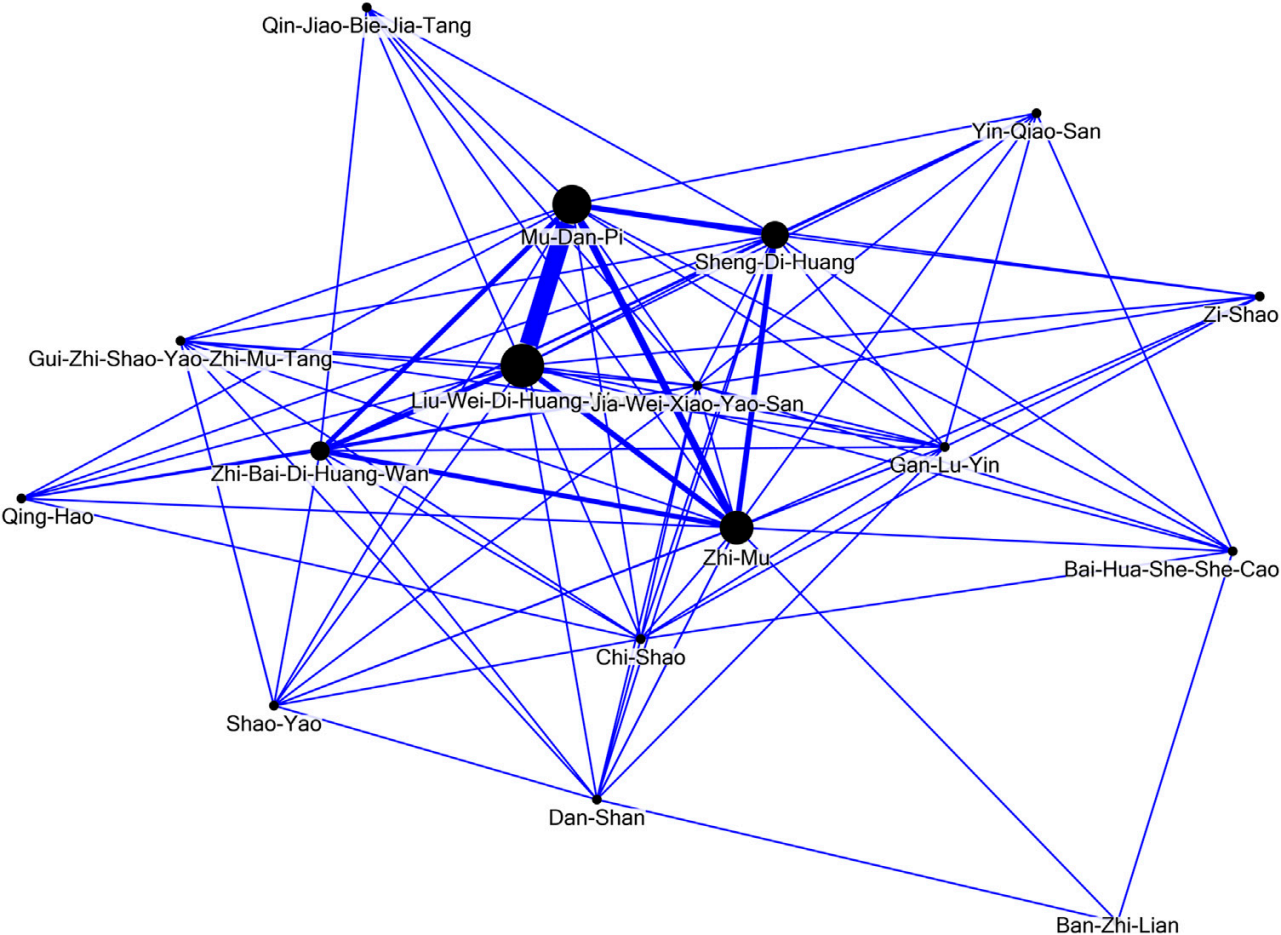
Network analysis of the seventeen potentially renoprotective Chinese herbal medicines for *systemic lupus erythematosus* (PRCHMSLE).

The network analysis (Figure 4) revealed the dominant patterns of both formulas and individual herbs used in the treatment of SLE. The core patterns comprised Liu-Wei-Di-Huang-Wan, *Paeonia suffruticosa* Andr. [Paeoniaceae; Cortex Moutan] (Mu-Dan-Pi), *Anemarrhena asphodeloides* Bunge [Asparagaceae; Rhizoma Anemarrhenae] (Zhi-Mu), *Rehmannia glutinosa* (Gaertn.) DC. [Orobanchaceae; Radix Rehmanniae] (Sheng-Di-Huang), and Zhi-Bai-Di-Huang-Wan.

### 3.6 Sensitivity analyses

We conducted three sensitivity analyses to verify the robustness of our results. In the subgroup analyses of patients with SLE-CKD, the outcomes for ESRD and all-cause mortality were predominantly favorable, and this trend persisted across most subgroups in both the Cox and RMST analyses, with a preference towards PRCHMSLE use over nonuse (Supplementary Table S2). When excluding patients with SLE-CKD who either died or developed ESRD within 30, 60, or 90 days post the index date, these associations remained consistent (Supplementary Table S3). The relationship between PRCHMSLE use and the decreased risks of ESRD and all-cause mortality persisted regardless of the varying definitions of PRCHMSLE use among patients with SLE-CKD (Supplementary Table S4).

## 4 Discussion

This study is the first to highlight the main renal and survival (especially, non-cardiovascular survival) benefits of using 17 PRCHMSLE in patients with SLE-CKD. Moreover, PRCHMSLE use not only resulted in the reduction of hospitalization risk but also demonstrated no elevated likelihood of hyperkalemia. These results build on prior research indicating renal and survival advantages (Chang et al., 2017) and remain consistent across multiple subgroups, further solidifying their validity through extensive sensitivity analyses. The findings suggest the targeting of various pathways associated with inflammation and oxidative stress for immune modulation in SLE. Notably, the top five treatments offering the most renal protection were Gan-Lu-Ying, *Anemarrhena asphodeloides* Bunge [Asparagaceae; Rhizoma Anemarrhenae] (Zhi-Mu), *Rehmannia glutinosa* (Gaertn.) DC. [Orobanchaceae; Radix Rehmanniae] (Sheng-Di-Huang), Jia-Wei-Xiao-Yao-San, and *Paeonia suffruticosa* Andr. [Paeoniaceae; Cortex Moutan] (Mu-Dan-Pi). Furthermore, this research introduces a groundbreaking discovery: a core prescription pattern, identified through network analysis that features Liu-Wei-Di-Huang-Wan, *Paeonia suffruticosa* Andr. [Paeoniaceae; Cortex Moutan] (Mu-Dan-Pi), *Anemarrhena asphodeloides* Bunge [Asparagaceae; Rhizoma Anemarrhenae] (Zhi-Mu), *Rehmannia glutinosa* (Gaertn.) DC. [Orobanchaceae; Radix Rehmanniae] (Sheng-Di-Huang), and Zhi-Bai-Di-Huang-Wan.

SLE is a complex disease affecting multiple bodily systems, including renal, cardiovascular, and pulmonary functions (Vymetal et al., 2016). Recent cohort studies using Taiwan’s NHI claims data have spotlighted potential benefits of CHMs in SLE management, indicating decreased risks of LN (Chang et al., 2017), mortality (Ma et al., 2016), cardiovascular disease (Yu and Hsieh, 2021), pneumonia (Wang et al., 2023), and hospitalization (Ma et al., 2023). Significantly, LN is a chief factor in SLE-related deaths (Sciascia et al., 2017). A notable study by Chang et al. (Chang et al., 2017) emphasized that combining conventional and herbal medicine therapies reduced the risk of LN among Taiwanese SLE patients. Ma et al. (Ma et al., 2016) pinpointed various CHM formulas, such as Zhi-Bo-Di-Huang-Wan, Jia-Wei-Xiao-Yao-San, and Liu-Wei-Di-Huang-Wan, which markedly improved patient survival. However, our research opinions distinguish themselves from prior studies in several key aspects. Firstly, earlier investigations utilizing NHI claims data typically juxtaposed overall CHM users with non-users, rather than specifically delving into the 17 PRCHMSLE identified as suitable for SLE treatment. Secondly, all preceding studies extracted the top ten medications used post CHM application from the NHI claims data and subsequently examined each drug’s mechanism within the context of SLE. Notably, they did not independently analyze CHMs known to provide protection against renal implications and SLE, marking a significant point of departure. Thirdly, no previous studies undertook a pooling effect or network analysis on the 17 PRCHMSLE deemed appropriate for SLE, underscoring a unique aspect of our research approach. Fourthly, this study represents a pioneering effort in evaluating the safety, particularly the risk of hyperkalemia, in patients with SLE-CKD undergoing treatment with CHMs, with a specific focus on PRCHMSLE. Fifthly, this study represents the inaugural application of RMST analysis in research involving CHM and SLE, offering valuable insights and more interpretable results than hazard ratios for assessing the clinical efficacy of PRCHMSLE. Our findings indicated that PRCHMSLE use was associated with a postponement of ESRD by 0.57 years, a delay in mortality by 1.22 years, and a deferment of non-cardiovascular events by 1.21 years over a 15-year period.

In line with previous research (Ma et al., 2016; Chen et al., 2022; Chen et al., 2023; Ma et al., 2023), our findings indicated that patients receiving the 17 PRCHMSLE may experience a reduction in all-cause mortality, a lower incidence of ESRD, fewer hospital admissions, and no potential for an increase in the risk of hyperkalemia. We also identified a lower risk of non-cardiovascular mortality in patients with SLE-CKD using PRCHMSLE compared to nonusers, mirroring findings from a previous NHI-based cohort study that indicated a reduced risk of pneumonia in patients with SLE using CHMs compared to nonusers (Wang et al., 2023). However, our results did not demonstrate a significant reduction in the risk of cardiovascular mortality in patients with SLE-CKD using PRCHMSLE. This contrasted with a previous NHI-based cohort study (Yu and Hsieh, 2021) that suggested a lower cardiovascular disease risk in patients with SLE who were on CHM. The differences in event rates observed in distinct study populations, such as those with SLE (Yu and Hsieh, 2021) *versus* SLE-CKD in our study, might contribute to the observed disparity.

Historically, research utilizing Taiwan’s NHI claims data primarily addressed prescription frequencies for a range of medical conditions. Yet, this data might not truly mirror underlying prescription patterns or therapeutic objectives, potentially causing misconceptions in future research and clinical trials. Using network analysis alongside data mining offers a deeper understanding of the clinical reasoning and prevalent agreement among traditional Chinese medicine experts. This underlines the need to weave evidence-based methods into upcoming CHM research. Our prior study (Chang et al., 2017) employed network analysis to identify main CHM prescription patterns for SLE, aligning with frequently prescribed medicines. In the present study, network analysis was employed to unravel the predominant prescription patterns for the treatment of patients with SLE. This methodology was tailored to evaluate the selected dual-drug combinations. In this context, connections between CHM and their co-prescribed counterparts were symbolized, with a denser line width denoting more frequent prescription affiliations. Within the 17 PRCHMSLE, network core pattern analysis identified the top five with renoprotective qualities as Gan-Lu-Ying, *Anemarrhena asphodeloides* Bunge [Asparagaceae; Rhizoma Anemarrhenae] (Zhi-Mu), *Rehmannia glutinosa* (Gaertn.) DC. [Orobanchaceae; Radix Rehmanniae] (Sheng-Di-Huang), Jia-Wei-Xiao-Yao-San, and *Paeonia suffruticosa* Andr. [Paeoniaceae; Cortex Moutan] (Mu-Dan-Pi). These quintessential formulae and individual herbs, recurrently prescribed in our study, exhibit antioxidant, anti-inflammatory, and immune-modulatory properties (Ma et al., 2016; Zhao et al., 2016; Zhuang et al., 2022).

Managing SLE and CKD necessitates a holistic approach targeting autoimmune responses, oxidative stress, inflammation, and fibrosis (Davidson, 2016; Bona et al., 2020). These 17 PRCHMSLE collectively display antioxidant, anti-inflammatory, and antifibrotic characteristics that combat renal failure (Huang et al., 2016; Jiang et al., 2020; Wang et al., 2021). Gan-Lu-Yin, historically revered in ancient Asia, is recognized for treating oral inflammations such as periodontitis, stomatitis, and glossodynia (Inagaki et al., 2021). In terms of autoimmunity, Gan-Lu-Yin has shown notable protective effects on SLE patient survival (Ma et al., 2016). It acts as an intervention, curbing T cell activation and reducing TH responses, as seen in Sjögren’s syndrome (Lee et al., 2021). Furthermore, it deters TNF-α expression in human oral cancer cells through the ERK and NF-κB pathways (Yang et al., 2016). These insights infer that Gan-Lu-Yin extracts bear anti-inflammatory qualities and act as inhibitors of tissue degradation. Studies indicate that *Anemarrhena asphodeloides* Bunge [Asparagaceae; Rhizoma Anemarrhenae] (Zhi-Mu) derivatives exhibit a plethora of pharmacological activities, encompassing antioxidation (Zhao et al., 2016), anti-inflammatory responses (Liu et al., 2023), antidiabetic properties (Yuan et al., 2015), and immune modulation (Zhang et al., 2020). *Rehmannia glutinosa* (Gaertn.) DC. [Orobanchaceae; Radix Rehmannia (Sheng-Di-Huang), esteemed for its capacity to purge pathogenic heat from blood and enrich Yin per Chinese medical doctrine (Zhang et al., 2008), is prevalent in many SLE prescriptions like Liu-Wei-Di-Huang-Wan and Zhi-Bai-Di-Huang-Wan (Ma et al., 2016; Chang et al., 2017). Its primary active element has proven to activate the Nrf2/Keap1 route and suppress pro-inflammatory factor expression, thus assuming dual roles as anti-inflammatory and antioxidant agents (Ren et al., 2023). Sheng-Di-Huang’s (*Rehmannia glutinosa* (Gaertn.) DC. [Orobanchaceae; Radix Rehmannia) therapeutic facets may be rooted in its anti-inflammatory and immunomodulatory effects, likely derived from its multifaceted composition engaging various targets and channels (Wang et al., 2022; Zhuang et al., 2022). *Paeonia suffruticosa* Andr. [Paeoniaceae; Cortex Mouta (Mu-Dan-Pi) extracts have demonstrated inhibitory influences on NF-κB and IRF reporters, along with curtailing downstream cytokine production, behaving as dose-regulated immunomodulators (Chen et al., 2020). Moreover, they offer antioxidant potency and present anti-inflammatory properties (Li et al., 2018). *Paeonia suffruticosa* Andr. [Paeoniaceae; Cortex Mouta (Mu-Dan-Pi) is integral to Jia-Wei-Xiao-Yao-San, a commonly prescribed formula in Taiwan. It is notable that Jia-Wei-Xiao-Yao-San also features prominently in Taiwan’s NHI claims data, largely because of its broad therapeutic applications for ailments like sleep disorders and depression (Chen et al., 2015b)—ailments frequently concurrent with SLE. This concoction, classified under harmonizing formula prescription patterns, is acknowledged for its renoprotective traits in kidney diseases (Lin et al., 2021). Additionally, it showcases antifibrotic and antioxidative qualities, including the suppression of xanthine oxidase activity in hepatic fibrosis rats (Chien et al., 2014).

The study boasts significant strengths. It utilized a nationally representative sample, guaranteeing strong statistical power and dependability. Comprehensive tracking of study events and prescriptions reduced information and recall bias. Several statistical methods were adopted, including propensity score matching to counteract confounding, competing risk analysis to avert ESRD overestimation, RMST analysis for straightforward estimates, and sensitivity analyses to ensure result dependability and accuracy. Additionally, in-depth assessments of the pooling effect and network analysis concerning renal and survival outcomes were conducted for the seventeen PRCHMSLE.

However, the study also presents certain limitations. First, it did not assess adherence to prescribed PRCHMSLE or potential interactions between herbal and Western medicines. Moreover, it overlooked pulse and syndromic diagnoses present in administrative claims. Second, care should be taken when generalizing these findings to Western healthcare settings since the PRCHMSLE formulas are sanctioned by Taiwan’s Committee of Chinese Medicine and Pharmacy. Third, even after employing propensity score matching and adjusting variables in the Cox proportional hazard regression, certain confounders absent from LGTD 2005, like lifestyle, health behaviors, environmental factors, and specific laboratory data, restrict the all-encompassing evaluation of ESRD and mortality risks. Fourth, as our analysis was solely centered on CHM, we did not present or delve into other alternative modalities such as acupuncture, tuina, massage, or those documented in the LGTD 2005. Lastly, being an observational study, it does not ascertain causation, and unaccounted-for confounding factors might affect the results.

## 5 Conclusion

Our real-world results show significant renal and survival advantages without an associated increase in hospitalization and hyperkalemia risks linked to the use of seventeen PRCHMSLE in treating patients with SLE-CKD. These herbal medicines have demonstrated marked renoprotective effects for SLE-CKD patients by modulating various targets within oxidative stress and inflammation pathways. Additionally, our network analysis has unveiled the core prescription pattern, underscoring the potential for diverse therapeutic strategies and alternative/complementary approaches in SLE-CKD management, thus broadening the scope for disease management.

## Data Availability

The raw data supporting the conclusion of this article will be made available by the authors, without undue reservation.

## Abbreviations

SLE, systemic lupus erythematosus; LN, lupus nephritis; ESRD, end-stage renal disease; CKD, chronic kidney disease; CHM, Chinese herbal medicine; NHI, National Health Insurance; PRCHMSLE, potentially renoprotective CHMs for systemic lupus erythematosus; LGTD2005; Longitudinal Generation Tracking Database; RMST, restricted mean survival time.
