# Supplementary material for: Renal and survival benefits of seventeen prescribed Chinese herbal medicines against oxidative-inflammatory stress in systemic lupus erythematosus patients with chronic kidney disease: a real-world longitudinal study
Source: Front Pharmacol. 2024 Jan 3;14:1309582. doi: 10.3389/fphar.2023.1309582 (PMC10791791; doi:10.3389/fphar.2023.1309582)

| **Supplemental Table 1**. The ingredients of 17 potentially renoprotective Chinese herbal medicines forSLE (PRCHMSLE) | |
| --- | --- |
| 17 PRCHMSLE | Ingredients or Generic Name |
| Sheng-Di-Huang  Zhi-Mu  Bai Shao  Mu-Dan-Pi  Dan-Shan  Chi-Shao  Zi-Cao  Qing-Hao  Bai-Hua-She-She-Cao  Ban-Zhi-Lian  Zhi-Bai-Di-Huang-Wan  Liu-Wei-Di-Huang-Wan  Gan-Lu-Ying  Qin-Jiao-Bie-Jia-Tang  Jia-Wei-Xiao-Yao-San  Yin-Qiao-San  Gui-Zhi-Shao-Yao-ZhiMu-Tang | *Rehmanniaglutinosa*Libosch (raw Rehmannia)  *Anemarrhenaasphodeloides*Bunge (RhizomaAnemarrhenae)  *Paeonia lactiflora*Pall. (Radix Paeoniae Alba)  *Paeonia suffruticosa* Andr.  *Salvia miltiorrhiza*Bge.  *Paeonia lactiflora* Rubra  *Lithospermum erythrorhizon* Siebold &Zucc.  *Artemisia annua* L.  *Hedyotisdiffusa* Willd (H. diffusa)  *Scutellariabarbata*D.Don  Zhi-Mu (RhizomaAnemarrhenae; *Anemarrhenaasphodeloides* Bung), Huang-Bo (PhellodendronchinenseC.K.Schneid.), Sheng-Di-Huang (raw *Rehmanniaglutinosa*Libosch.), Shan-Zhu-Yu (*Cornus officinalis* Siebold &Zucc.), Mu-Dan-Pi (*Paeonia suffruticosa* Andr.), Shan-Yao (*Dioscorea japonica* Thunb.), Fu-Ling (Poria; *Wolfiporiacocos* (Schw.)), Ze-Xie (RhizomaAlismatis; *Alisma orientale* (Sam.) Juz.).  Sheng-Di-Huang (raw *Rehmanniaglutinosa*Libosch.), Shan-Zhu-Yu (*Cornus officinalis* Siebold &Zucc.), Mu-Dan-Pi (*Paeonia suffruticosa* Andr.), Shan-Yao (*Dioscorea japonica* Thunb.), Fu-Ling (Poria; *Wolfiporiacocos* (Schw.)), Ze-Xie (RhizomaAlismatis; *Alisma orientale* (Sam.) Juz.).  Di-Huang (Radix Rehmanniae; *Rehmanniaglutinosa*(Gaertn.) DC.), Shi-Hu (HerbaDendrobii; *Dendrobium*  *loddigesii*Rolfe), Tian-Men-Dong (Radix Asparagi; *Asparagus cochinchinensis* (Lour.) Merr.), Mai-Men-Dong (Radix Ophiopogonis; *Ophiopogon japonicus* (Thunb.) Ker Gawl.), Huang-Qin (Radix Scutellariae; *Scutellariabaicalensis* Georgi), Yin-Chen-Hao (HerbaArtemisiaeScopariae; *Artemisia capillaris*Thunb.), Zhi-Ke (Fructus Aurantii; *Citrus × aurantium* L.), Pi-Pa-Ye (Folium Eriobotryae; *Eriobotrya japonica* (Thunb.) Lindl.), Gan-Cao (Radix Glycyrrhizae; *Glycyrrhiza uralensis* Fisch.).  Di-Gu-Pi (Cortex Lycii; *Lyciumbarbarum* L.), Chai-Hu (Radix Bupleuri; *Bupleurum chinense* DC.), Qin-Jiao (*Gentiana macrophylla* Pall.), Zhi-Mu (RhizomaAnemarrhenae; *Anemarrhenaasphodeloides* Bung),  Dang-Gui (Radix Angelicae Sinensis; *Angelica sinensis* (Oliv.) Diels), Bie-Jia (CarapaxTrionycis; *Pelodiscus sinensis*), Wu-Mei (Mume Fructus; *Prunus mume* (Siebold) Siebold &Zucc.), Qing-Hao (*Artemisia annua* L.).  Dang-Gui (Radix Angelicae Sinensis; *Angelica sinensis* (Oliv.) Diels), Bai-Shao (Radix Paeoniae Alba; *Paeonia lactiflora* Pall.), Fu-Ling (Poria; *Wolfiporiacocos* (Schw.) Ryv. and Cilbn.), Bai-Zhu (RhizomaAtractylodisMacrocephalae; *Atractylodesmacrocephala*Koidz.), Chai-Hu (Radix Bupleuri; *Bupleurum chinense* DC.), Mu-Dan-Pi (Cortex Moutan; *Paeonia × suffruticosa* Andrews), Zhi-Zi (Fructus Gardeniae; *Gardenia jasminoides*J.Ellis), Gan-Cao (Radix Glycyrrhizae; *Glycyrrhiza uralensis*Fisch.), Bo-He (HerbaMenthaeHaplocalycis; *Mentha alaica* Boriss.), Sheng-Jiang (RhizomaZingiberisRecens; *Zingiber officinale* Roscoe).  Jin-Yin-Hua(*Lonicera japonica* Thunb),Lien-Chiao (*Forsythia suspensa*(Thunb.) Vahl), Jie-Geng(*Platycodon grandiflorum* (Jacq.) A.DC), Niu-Bang-Zi (*Arctium lappa* L. Fruit (Arctii Fructus)), Bo-He (*Mentha haplocalyx* Briq.), Dan-Dou-Chi (*Glycine max* (L.) Merr.), Dan-Zhu-Ye (*Lophatherum*  *gracile* Bronghiart (*L. gracile*)), Jing-Jie (HerbaSchizonepetae; *Schizonepeta tenuifolia* Briq.), Huang-Lien (*Coptis chinensis* Franch.), Gan-Cao (RadixGlycyrrhizae; *Glycyrrhiza uralensis* Fisch.).  Gui-Zhi (RamulusCinnamomi; *Cinnamomum cassia*(L.) J.Presl), Ma-Huang (HerbaEphedrae; *Ephedra sinica* Stapf), Fu-Zi (Radix Aconiti Lateralis; *Aconitum carmichaeli*var. carmichaeli), Zhi-Mu (RhizomaAnemarrhenae; *Anemarrhenaasphodeloides* Bunge), Bai-Shao (Radix Paeoniae Alba; *Paeonia lactiflora* Pall.), Bai-Zhu (RhizomaAtractylodisMacrocephalae; *Atractylodesmacrocephala*Koidz.), Fang-Feng (Radix Saposhnikoviae; *Saposhnikoviadivaricata* (Turcz.) Schischk.), Sheng-Jiang (RhizomaZingiberisRecens; *Zingiber officinale* Roscoe), Gan-Cao (Radix Glycyrrhizae; *Glycyrrhiza uralensis* Fisch.) |

| **Supplemental Table 2.** Subgroup analyses of patients with SLE-CKD by Cox and RMST analyses | | | | | | | | | | | | | | | |
| --- | --- | --- | --- | --- | --- | --- | --- | --- | --- | --- | --- | --- | --- | --- | --- |
|  | ESRD | | | | | | |  | Overall mortality | | | | | | |
|  | Use (*vs.* Nonuse) | | |  | Use (*vs.* Nonuse) | | |  | Use (*vs.* Nonuse) | | |  | Use (*vs.* Nonuse) | | |
| Variable | aHR | 95% CI | *p*-value |  | RMSTd (y) | 95% CI | *p*-value |  | aHR | 95% CI | *p*-value |  | RMSTd (y) | 95% CI | *p*-value |
| Sex |  |  |  |  |  |  |  |  |  |  |  |  |  |  |  |
| Male | 1.68 | 0.28-10.19 | 0.57 |  | -0.48 | -2.03-1.08 | 0.55 |  | 0.73 | 0.35-1.53 | 0.40 |  | 0.64 | -1.31-2.58 | 0.52 |
| Female | 0.40 | 0.20-0.81 | 0.011 |  | 0.72 | 0.22-1.21 | 0.0045 |  | 0.44 | 0.30-0.64 | <0.0001 |  | 1.50 | 0.77-2.23 | <0.0001 |
| Age (year) |  |  |  |  |  |  |  |  |  |  |  |  |  |  |  |
| ≤35 | 0.21 | 0.07-0.63 | 0.006 |  | 1.73 | 0.74-2.72 | 0.0006 |  | 0.52 | 0.25-1.07 | 0.07 |  | 1.06 | -0.11-2.24 | 0.08 |
| >35 | 0.97 | 0.41-2.29 | 0.95 |  | 0.01 | -0.48-0.51 | 0.96 |  | 0.48 | 0.33-0.70 | 0.0001 |  | 1.51 | 0.68-2.33 | 0.0003 |
| Diabetes |  |  |  |  |  |  |  |  |  |  |  |  |  |  |  |
| No | 0.50 | 0.26-0.95 | 0.036 |  | 0.62 | 0.13-1.11 | 0.013 |  | 0.44 | 0.30-0.63 | <0.0001 |  | 1.60 | 0.91-2.30 | <0.0001 |
| Yes | 1.04 | 0.04-26.27 | 0.98 |  | 0 | -1.11-1.12 | 1.00 |  | 0.61 | 0.23-1.64 | 0.32 |  | -0.15 | -2.82-2.52 | 0.91 |
| Hypertension |  |  |  |  |  |  |  |  |  |  |  |  |  |  |  |
| No | 0.62 | 0.24-1.60 | 0.32 |  | 0.24 | -0.36-0.84 | 0.44 |  | 0.50 | 0.27-0.93 | 0.029 |  | 0.85 | 0.03-1.66 | 0.042 |
| Yes | 0.42 | 0.18-1.01 | 0.05 |  | 1.03 | 0.28-1.78 | 0.007 |  | 0.48 | 0.33-0.72 | 0.0003 |  | 1.95 | 0.87-3.02 | 0.0004 |
| Hyperlipidemia |  |  |  |  |  |  |  |  |  |  |  |  |  |  |  |
| No | 0.42 | 0.21-0.82 | 0.012 |  | 0.73 | 0.23-1.23 | 0.004 |  | 0.45 | 0.31-0.65 | <0.0001 |  | 1.51 | 0.79-2.22 | <0.0001 |
| Yes | 2.21 | 0.27-18.22 | 0.46 |  | -0.7 | -2.41-1.01 | 0.42 |  | 0.50 | 0.19-1.32 | 0.16 |  | 0.83 | -1.43-3.08 | 0.47 |
| Rheumatoid arthritis |  |  |  |  |  |  |  |  |  |  |  |  |  |  |  |
| No | 0.50 | 0.27-0.95 | 0.033 |  | 0.59 | 0.11-1.07 | 0.016 |  | 0.48 | 0.35-0.67 | <0.0001 |  | 1.41 | 0.71-2.10 | <0.0001 |
| Yes | Not converged | | |  | 0 | 0-0 | - |  | Not converged | | |  | -0.87 | -3.56-1.82 | 0.53 |
| Sjögren's syndrome |  |  |  |  |  |  |  |  |  |  |  |  |  |  |  |
| No | 0.54 | 0.29-1.02 | 0.06 |  | 0.53 | 0.05-1.00 | 0.029 |  | 0.50 | 0.36-0.70 | <0.0001 |  | 1.31 | 0.61-2.01 | 0.0002 |
| Yes | Not converged | | |  | 1.61 | -0.3-3.53 | 0.09 |  | Not converged | | |  | 1.80 | -1.82-5.43 | 0.33 |
| Raynaud's disease |  |  |  |  |  |  |  |  |  |  |  |  |  |  |  |
| No | 0.53 | 0.28-1.00 | 0.049 |  | 0.54 | 0.07-1.00 | 0.024 |  | 0.49 | 0.35-0.69 | <0.0001 |  | 1.32 | 0.63-2.01 | 0.0002 |
| Yes | Not converged | | |  | 6.41 | 3.89-8.93 | <0.0001 |  | Not converged | | |  | 1.98 | -0.56-4.51 | 0.13 |
| NSAID |  |  |  |  |  |  |  |  |  |  |  |  |  |  |  |
| No | 1.02 | 0.28-3.66 | 0.98 |  | 0.03 | -1.27-1.33 | 0.96 |  | 0.93 | 0.44-1.96 | 0.86 |  | 0.28 | -1.26-1.82 | 0.72 |
| Yes | 0.38 | 0.17-0.86 | 0.02 |  | 0.69 | 0.20-1.17 | 0.006 |  | 0.41 | 0.28-0.60 | <0.0001 |  | 1.65 | 0.89-2.41 | <0.0001 |
| Steroid |  |  |  |  |  |  |  |  |  |  |  |  |  |  |  |
| No | 0.53 | 0.28-0.99 | 0.047 |  | 0.56 | 0.08-1.03 | 0.021 |  | 0.51 | 0.36-0.71 | <0.0001 |  | 1.29 | 0.60-1.98 | 0.0003 |
| Yes | Not converged | | |  | 0.29 | -0.26-0.84 | 0.31 |  | Not converged | | |  | 0.74 | -0.34-1.82 | 0.18 |
| Drugs other than steroids |  |  |  |  |  |  |  |  |  |  |  |  |  |  |  |
| No | 0.50 | 0.27-0.95 | 0.033 |  | 0.57 | 0.1-1.05 | 0.017 |  | 0.49 | 0.35-0.69 | <0.0001 |  | 1.34 | 0.66-2.03 | 0.0001 |
| Yes | Not converged | | |  | 0 | 0-0 | - |  | Not converged | | |  | 1.64 | -0.44-3.71 | 0.12 |
| Charlson comorbidity index |  |  |  |  |  |  |  |  |  |  |  |  |  |  |  |
| <2 | 0.45 | 0.2-1.00 | 0.05 |  | 0.64 | 0.07-1.20 | 0.028 |  | 0.35 | 0.22-0.56 | <0.0001 |  | 1.76 | 0.99-2.54 | <0.0001 |
| ≥2 | 0.68 | 0.25-1.91 | 0.47 |  | 0.38 | -0.44-1.20 | 0.36 |  | 0.68 | 0.42-1.12 | 0.13 |  | 0.37 | -0.90-1.63 | 0.57 |
| Medical visits |  |  |  |  |  |  |  |  |  |  |  |  |  |  |  |
| ≤18 | 0.55 | 0.22-1.35 | 0.19 |  | 0.45 | -0.21-1.11 | 0.18 |  | 0.94 | 0.54-1.62 | 0.81 |  | 0.26 | -0.59-1.12 | 0.54 |
| >18 | 0.54 | 0.22-1.34 | 0.18 |  | 0.65 | 0.01-1.29 | 0.046 |  | 0.36 | 0.24-0.54 | <0.0001 |  | 2.32 | 1.27-3.36 | <0.0001 |
| Abbreviations: the same as Tables 1-3; RMSTd, RMST difference; y, year.  Adjusted for all covariates (age per year, sex, comorbidities, number of medical visits, Charlson comorbidity index, confounding drugs) and competing mortality for ESRD. | | | | | | | | | | | | | | | |

| **Supplemental Table 3.** Risks of study outcomes in SLE-CKD patients excluding dying or developing ESRD within 30, 60, and 90 days after the index date | | | | | | | | |
| --- | --- | --- | --- | --- | --- | --- | --- | --- |
|  |  |  | ESRD | |  |  | Overall mortality | |
|  |  | Event/N | RMST difference, year (95% CI) | aHR (95% CI) |  | Event/N | RMST difference, year (95% CI) | aHR (95% CI) |
| Follow-up  >30 days | Nonuse | 25/456 | 0 (Reference) | 1 (Reference) |  | 86/456 | 0 (Reference) | 1 (Reference) |
|  | Use | 15 / 464 | 0.57 (0.10, 1.04) | 0.50 (0.27, 0.94) |  | 66/464 | 1.39 (0.67, 2.10) | 0.50 (0.36, 0.69) |
| Follow-up  >60 days | Nonuse | 25/445 | 1 (Reference) | 1 (Reference) |  | 81/445 | 0 (Reference) | 1 (Reference) |
|  | Use | 15/462 | 0.58 (0.10, 1.05) | 0.49 (0.26, 0.93) |  | 66/462 | 1.25 (0.54, 1.96) | 0.54 (0.38, 0.76) |
| Follow-up  >90 days | Nonuse | 25/436 | 0 (Reference) | 1 (Reference) |  | 78/436 | 0 (Reference) | 1 (Reference) |
|  | Use | 15/459 | 0.58 (0.11, 1.05) | 0.49 (0.26, 0.93) |  | 66/459 | 1.17 (0.46, 1.88) | 0.53 (0.38, 0.74) |
| Abbreviations: the same as Tables 1-3.  Adjusted for all covariates (age per year, sex, comorbidities, number of medical visits, Charlson comorbidity index, confounding drugs), and competing risk for ESRD. | | | | | | | | |

| **Supplemental Table 4.** Study outcomes by definitions of PRCHMSLE use in SLE-CKD patients | | | | | | | | |
| --- | --- | --- | --- | --- | --- | --- | --- | --- |
|  |  |  | ESRD | |  |  | Overall mortality | |
|  |  | Event/Number | RMSTd  (y, 95% CI) | aHR  (95% CI) |  | Event/Number | RMSTd  (y, 95% CI) | aHR  (95% CI) |
| Over 30 days as use | |  |  |  |  |  |  |  |
| Nonuse | | 21/306 | 0 (Reference) | 1 (Reference) |  | 59/306 | 0 (Reference) | 1 (Reference) |
| Use | | 11/306 | 0.82 (0.24, 1.41) | 0.39 (0.19, 0.80) |  | 32/306 | 1.90 (1.12, 2.68) | 0.33 (0.21, 0.52) |
| Over 60 days as use | |  |  |  |  |  |  |  |
| Nonuse | | 19/244 | 0 (Reference) | 1 (Reference) |  | 49/244 | 0 (Reference) | 1 (Reference) |
| Use | | 10/244 | 0.99 (0.31, 1.67) | 0.38 (0.18, 0.80) |  | 26/244 | 2.03 (1.17, 2.89) | 0.30 (0.18, 0.50) |
| Abbreviation: the same as Tables 1-3.  Adjusted for all covariates (age per year, sex, comorbidities, number of medical visits, Charlson comorbidity index, confounding drugs) and competing risk for ESRD. | | | | | | | | |

| **Supplemental Table 5.** Hospitalization risk during follow-up | | |
| --- | --- | --- |
|  | Users  (n=594) | Nonusers  (n=594) |
| Number (%) | 115 (19.4%) | 142 (23.9%) |
| Person-years observed | 4985 | 3788 |
| Incidence rate per 1000 person-years | 23.1 | 37.5 |
| Adjusted HR^*^ (95% CI) | 0.72 (0.56, 0.92)^*^ | 1 (Reference) |
| Abbreviations: the same as Tables 1-2.  Adjusted for all covariates (age per year, sex, comorbidities, number of medical visits, Charlson comorbidity index, and confounding drugs).  ^*^*p*=0.009 | | |

| **Supplemental Table 6.** Hyperkalemia risk during follow-up | | |
| --- | --- | --- |
|  | Users  (n=594) | Nonusers  (n=5946) |
| Hyperkalemia from outpatient and inpatient summation | | |
| Events (%) | 19 (3.2%) | 17 (2.9%) |
| Person-years observed | 4444 | 3372 |
| Incidence rate per 1000 person-years | 4.3 | 5.0 |
| Adjusted incidence rate ratio^*^ (95% CI) | 0.74 (0.49, 1.10)^*^ | 1 (Reference) |
| Abbreviations: the same as Tables 1-2.  Adjusted for all covariates (age per year, sex, comorbidities, number of medical visits, Charlson comorbidity index, and confounding drugs).  ^*^*p*=0.13 | | |

**Supplemental Figure 1.** Propensity score distribution (A) before matching, (B) after matching.


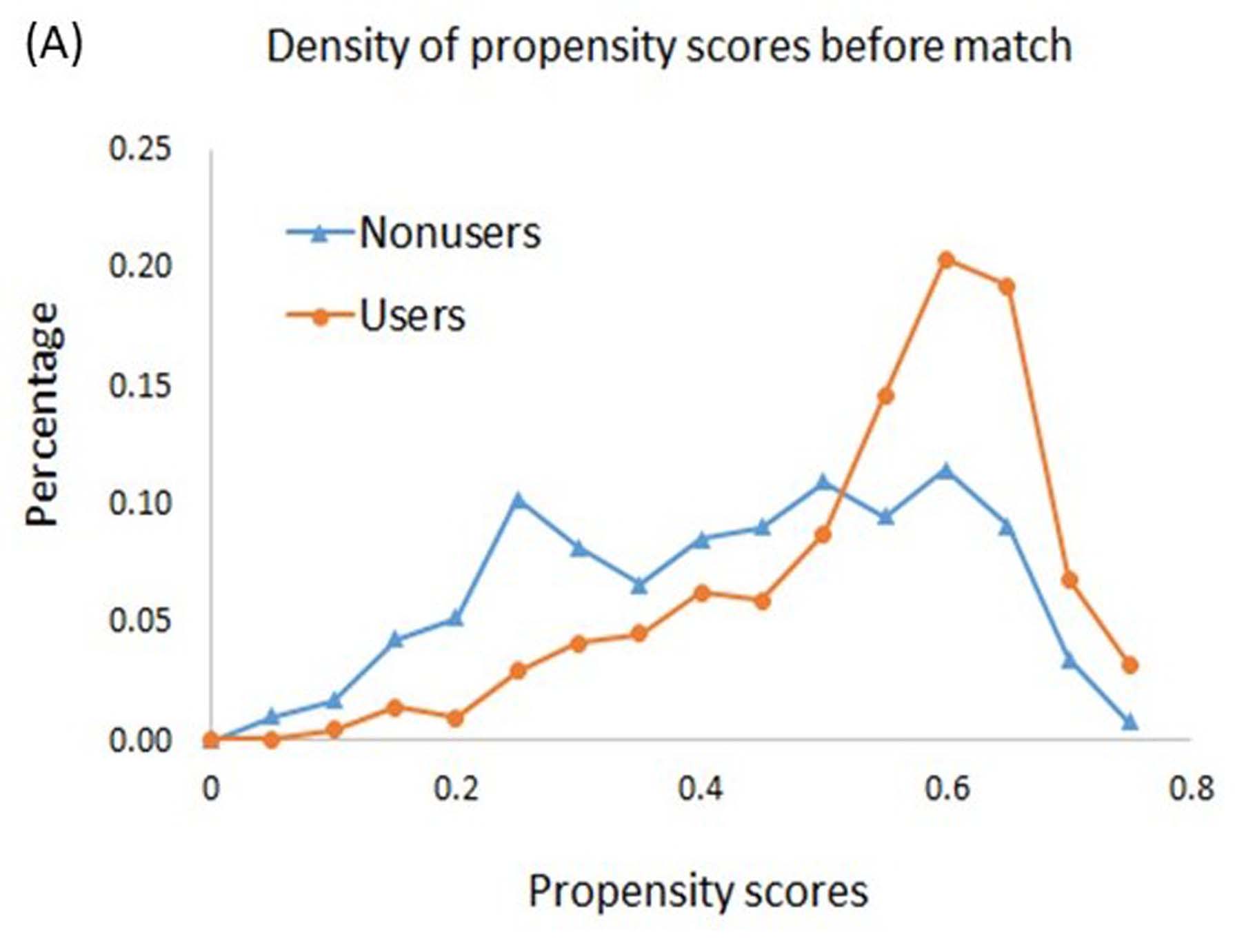


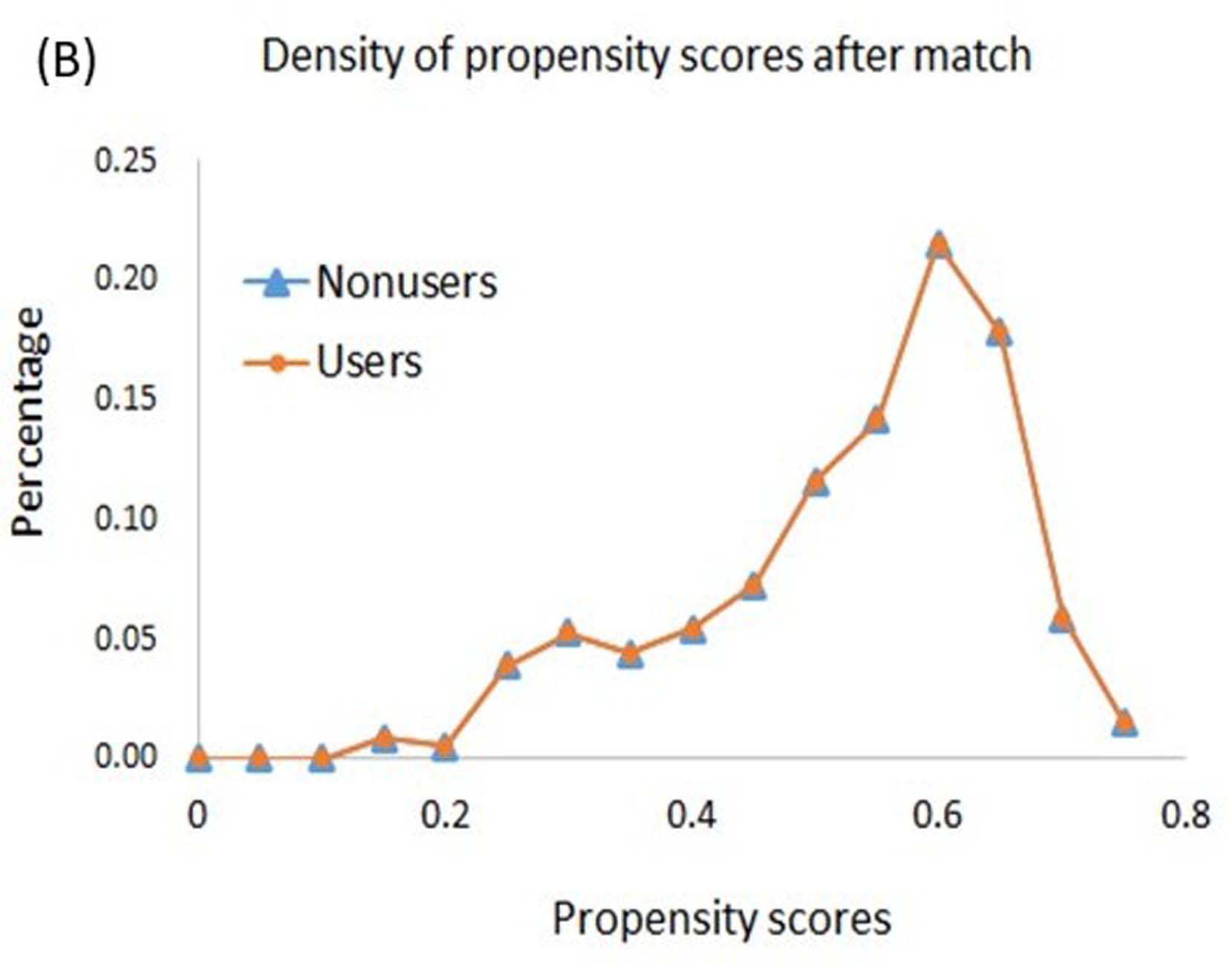

Supplement: Supplementary file 1 [file DataSheet1.doc]
